# Supplementary material for: Reward history guides focal attention in whisker somatosensory cortex
Source: Nat Commun. 2025 Jul 1;16:5580. doi: 10.1038/s41467-025-60592-w (PMC12219055; doi:10.1038/s41467-025-60592-w)
Supplement: Supplementary file 1 — Supplementary Information [file 41467_2025_60592_MOESM1_ESM.pdf]

Supplementary Figures

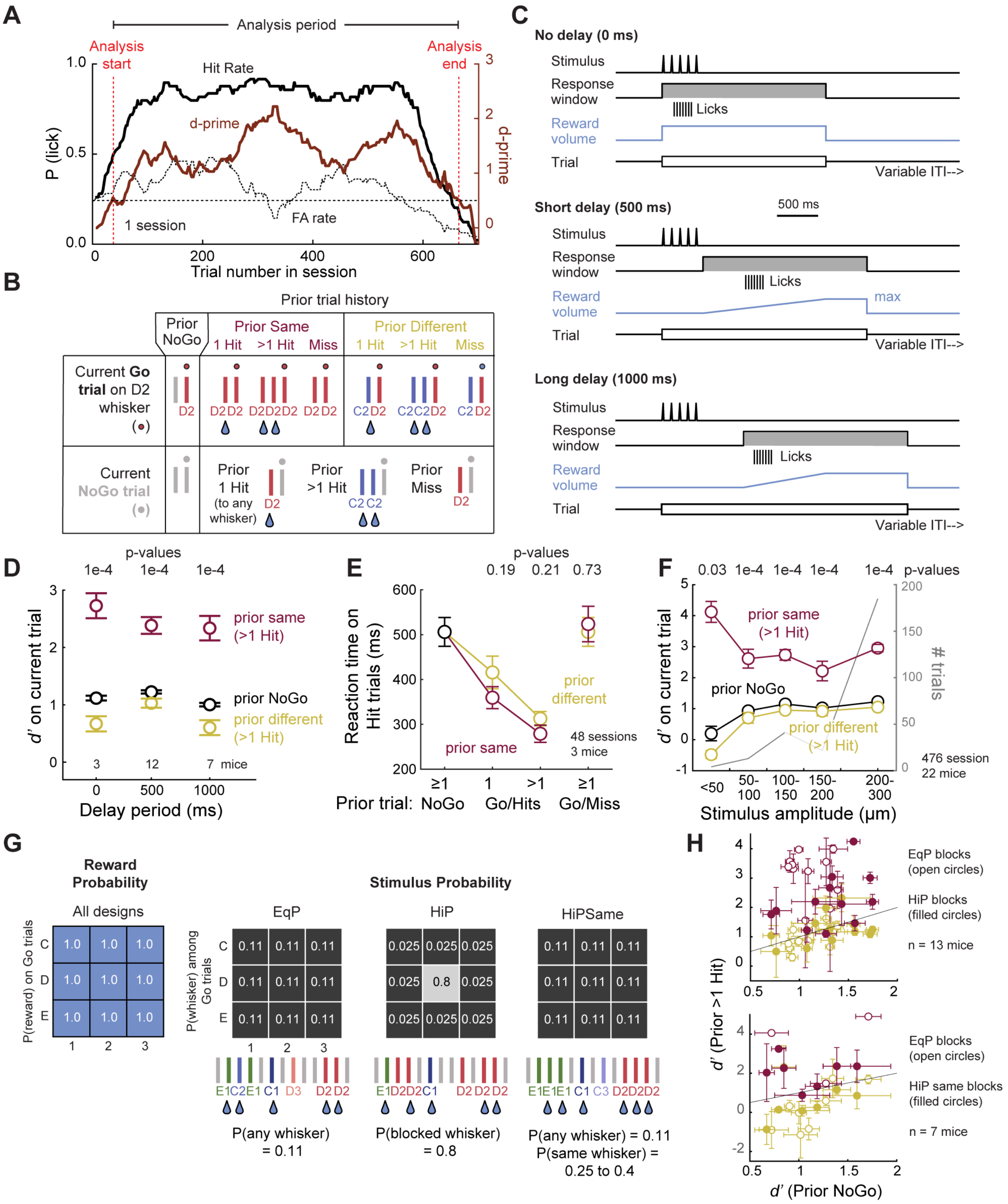

Figure S1. Further characterization of attentional capture by recent reward history. A. Behavioral performance in a single example session. Sliding *d′* was computed from Hit rate and False Alarm (FA) rate using a 50-trial sliding window. A *d′* cutoff (black dashed line) was applied to exclude trials at the start and end of the session. **B.** Definition of trial history classes for current Go trials and current NoGo trials, using current D2 whisker trials as an example. When calculating *d'* and *c*, Go and NoGo trials with matched trial histories were selected, as illustrated. **C.** Delay periods used for mice in different types of experiments. The 0 ms delay (no delay period) was used for mice that were only used for behavioral analyses . The 500 ms delay period was used for mice in 2p imaging and extracellular spike recording experiments. The 1000 ms delay period was used for mice in 2p

imaging experiments. For 500 ms and 1000 ms delay periods, reward volume was ramped during the delay period to encourage delayed licking (see Methods). **D.** Whisker-specific attentional cueing based on reward history was observed for mice trained with all 3 delay periods. (p = 1e-4 for prior same vs prior different for all delays, permutation test). **E.** Reaction times on Hit trials for different trial histories, for mice trained without a delay period. Prior hits decreased reaction time relative to prior NoGo (prior same >1: p = 1e-4, prior different >1 hit: p = 1e-4), but there was no significant difference between prior same and prior different conditions (prior same >1 hit vs prior different >1 hit: p = 0.21, permutation test). **F.** Trial history effect as a function of whisker stimulus amplitude (i.e., stimulus strength) on the current trial. This shows that history-dependent boosting in *d′* is strongest for low amplitude whisker stimuli (<50 μm: p = 0.03, 50-100 μm: p = 1e-4, 100-150 μm: p = 1e-4, 150-200 μm: p = 1e-4, 200-300 μm: p = 1e-4). P-values are for prior-same vs prior-different (permutation test). **G.** The three task designs used to set different whisker stimulus probabilities. In all designs, reward probability for a Hit on any whisker, P(reward), was 100%. In the equal probability (EqP) design, each of the 9 whiskers was presented with equal probability. In the high-probability (HiP) design, sessions were divided into one or more blocks of a few hundred trials, and within each block a single whisker (example, gray box) had a much higher probability (0.8) than the others (0.025). In the high probability of same whisker (HiPSame) design, all whiskers were presented at an equal overall probability, but sequential presentation was biased so that there was a higher probability (0.25-0.4) of the same whisker being repeated on the next trial, for all whisker identities. In all 3 designs, whisker identity was randomly selected on each Go trial, following the intended probability structure. **H.** Whisker-specific attentional effects cued by reward history were observed in all three task designs. 13 mice were tested with EqP and HiP designs (top), and 7 mice were tested with EqP and HiPSame designs (bottom). Related to **Figure 1** and **Figure 2**.

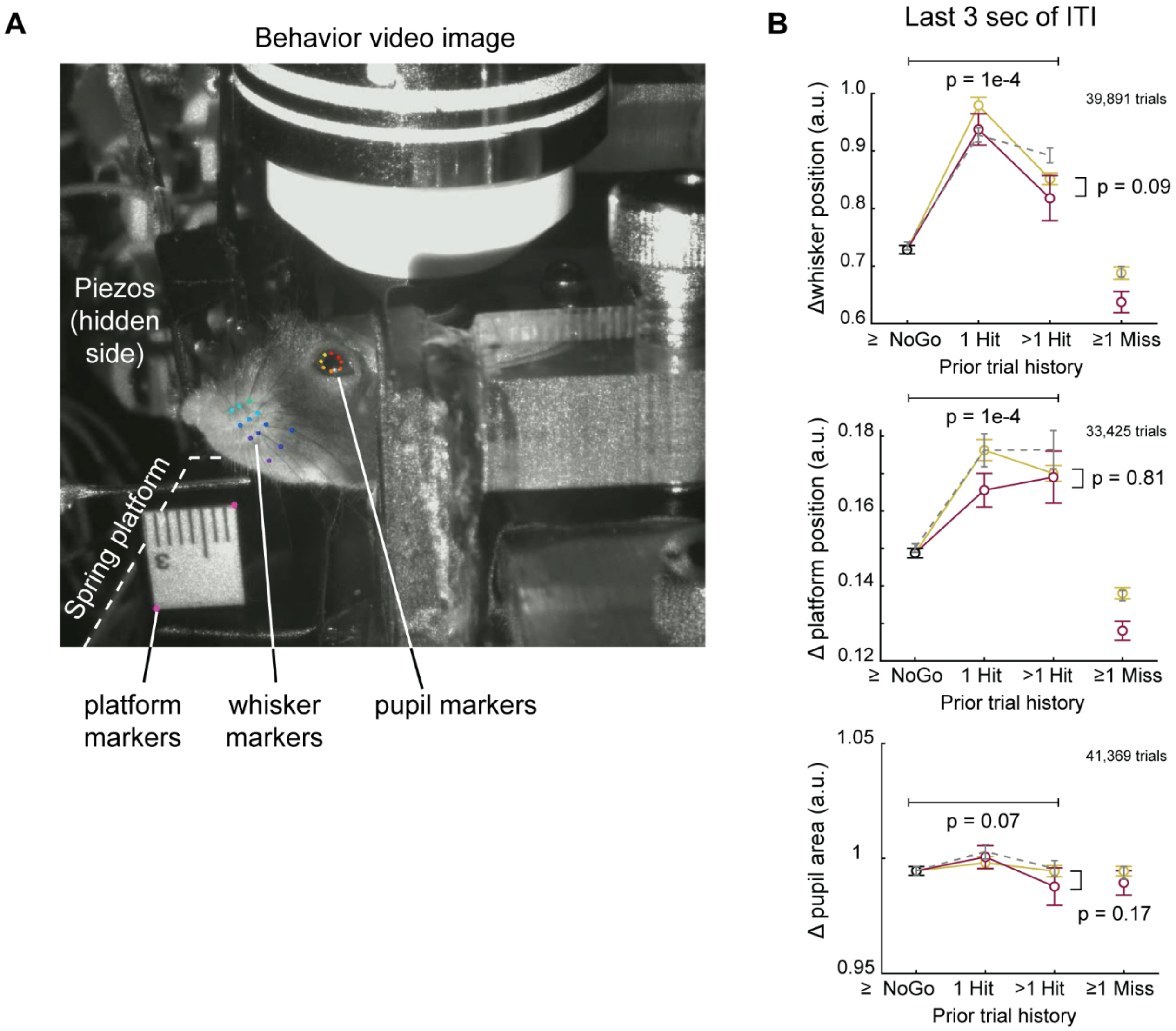

Figure S2. Behavioral imaging and additional analysis of history-dependent effects on spontaneous behaviors. **A.** Example behavioral movie frame showing DeepLabCut tracking of whisker motion (pad + 3 whiskers), platform motion (proxy for body motion), and pupil size measurement. **B.** Mean whisker motion, body motion, and pupil size changes during the ITI period of the current trial, as a function of trial history. Conventions and p-values as in **Fig. 3B**. Prior Hits increased average whisker and body motion during the intertrial interval (ITI) period (Δ whisker motion, prior >1 hit same vs prior NoGo: p = 1e-4, prior >1 hit same vs >1 hit different: p = 1e-4; Δ body motion, prior >1 hit same vs prior NoGo: p = 1e-4, prior >1 hit same vs >1 hit different: p = 0.55; Δ pupil area, prior >1 hit same vs prior NoGo: p = 0.07, prior >1 hit same vs >1 hit different: p = 0.16). Related to **Figure 3**.

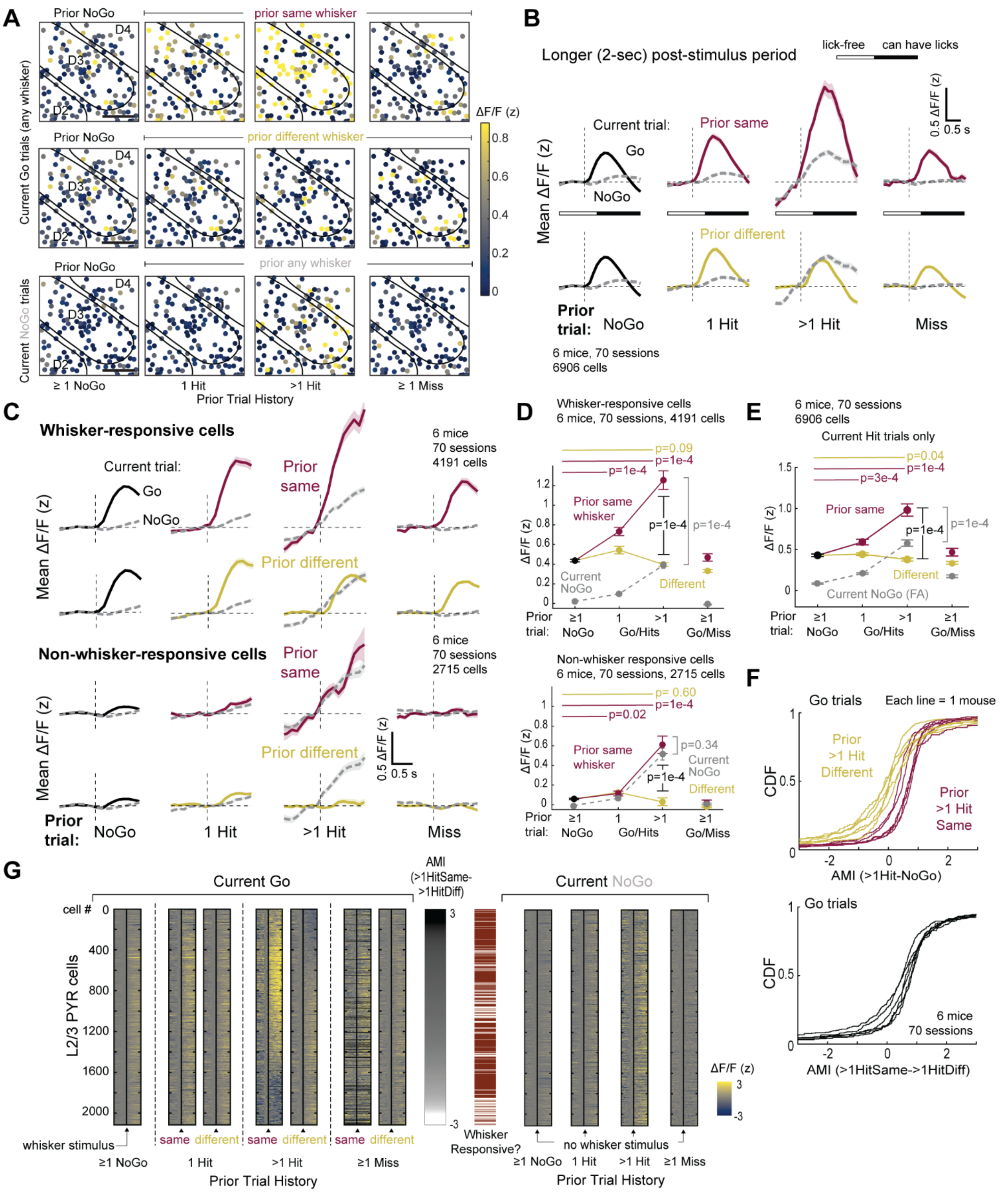

Figure S3. Additional analysis of history cueing effects on PYR activity. A. Example imaging field showing modulation of whisker-evoked response magnitude (color scale) by trial history. Top 2 rows, the average response to all Go whiskers. Response magnitude was increased for many cells in the prior Same >1 Hit condition, and not in the prior Different > 1 Hit condition. Bottom row, activity on NoGo trials, showing expectation or global arousal effect after > 1 prior Hit to any whisker. **B.** Mean whisker-evoked ΔF/F traces, as in **Fig. 3D**, but extending for 2 seconds post-stimulus. This includes both the lick-free post-stimulus period (0.7 sec, as shown in Fig. 3D), and an additional 1.3 sec in which there may be licks. Conventions as in **Fig 3D**. **C.** Mean whisker-evoked ΔF/F traces, as in **Fig. 3D**, calculated separately for whisker-responsive or non-responsive neurons (identified based on prior NoGo trials). **D.** Quantification of the data in (c). Conventions as in **Fig. 3E**.

Prior hits to the same whisker increase whisker-evoked responses in responsive cells (prior same 1 Hit vs prior NoGo: p = 1e-4, prior same >1 Hit vs prior NoGo: p = 1e-4, prior different >1 Hit vs prior NoGo: p = 0.09, prior same >1 Hit vs prior >1 Hit current NoGo: p = 1e-4, permutation test), but do not cause the appearance of whisker responses in non-responsive cells (prior same 1 Hit vs prior NoGo: p = 0.02, prior same >1 Hit vs prior NoGo: p = 1e-4, prior different >1 Hit vs prior NoGo: p = 0.60, prior same >1 Hit vs prior >1 Hit current NoGo: p = 0.34, permutation test). **E.** Same as Fig. 3E, but calculated only for current Hit and current FA trials. This analysis shows that the whisker-specific boosting of whisker-evoked DF/F represents increased sensory response gain, rather than an effect of behavioral decision (lick). Statistical tests are as in (**D**). **F.** CDFs of AMI for each cell, separated by mouse. The whisker-specific effect on response magnitude was observed in 6/6 mice. **G.** Mean ΔF/F trace for each L2/3 pyramidal (PYR cell), sorted by attention modulation index (AMI) $AMI_{>1HitSame->1HitDiff}$ for each cell. Related to **Figure 4** and **Figure S4**.

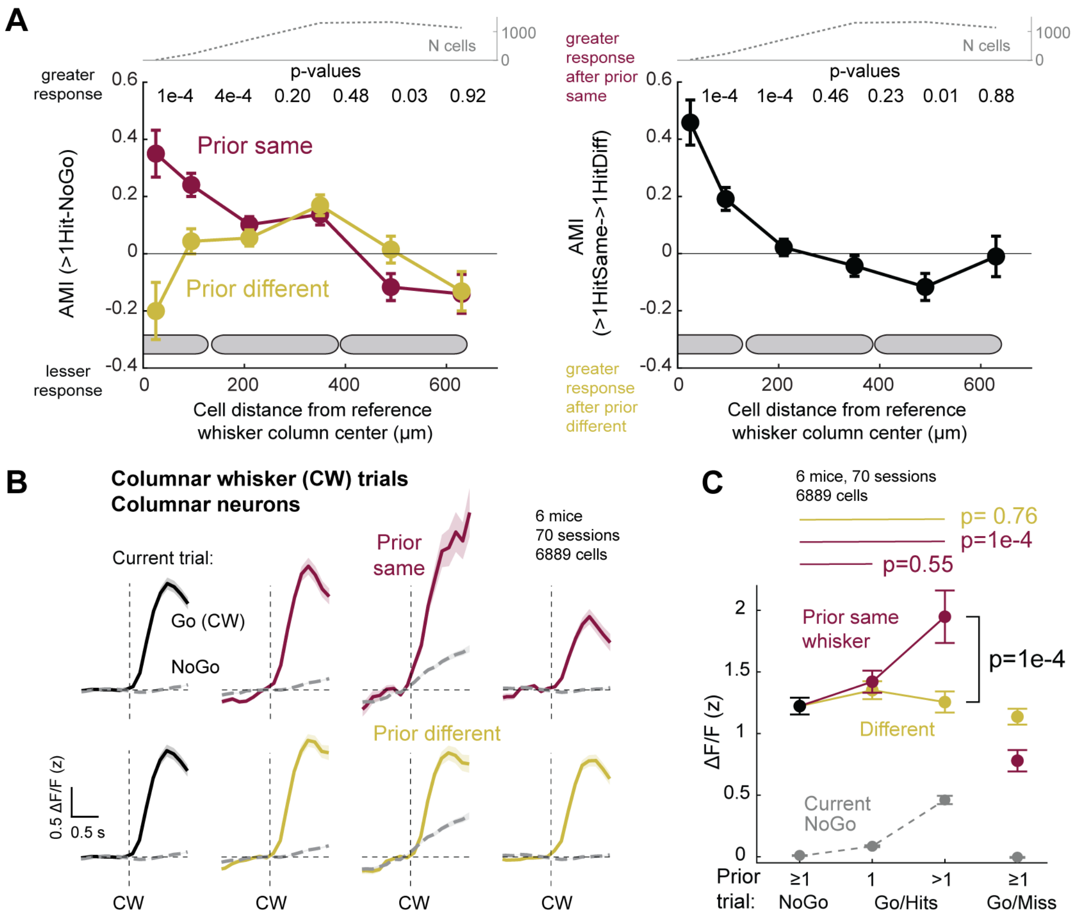

Figure S4. Additional analysis of somatotopic profile of attentional boosting in S1. A. Mean attention modulation index (AMI) for neuronal responses to a reference whisker, binned by cell location relative to the center of the reference whisker column. Left, $AMI_{>1HitSame-NoGo}$ and $AMI_{>1HitDiff-NoGo}$. Right, $AMI_{>1HitSame->1HitDiff}$. Conventions as in **Fig. 3K**, except that finer spatial bins are used within the reference whisker column. Whisker-specific attentional boosting was evident in a region <200 um from the reference column center ($AMI_{>1HitSame-NoGo}$ vs $AMI_{>1HitDiff-NoGo}$, 0 -50 μm: 1e-4, 50-140 μm: 4e-4,140-280 μm: 0.20, 280- 420 μm: 0.48, 420-560 μm: 0.03, 560-700 μm: 0.92, permutation test; $AMI_{>1HitSame->1HitDiff}$, 0 -50 μm: 1e-4, 50-140 μm: 1e-4,140-280 μm: 0.46, 280-420 μm: 0.23, 420-560 μm: 0.01, 560-700 μm: 0.88, one-sample permutation test vs mean of 0). **B-C.** Trial history-dependent modulation of neural responses to the columnar whisker (CW). For this analysis, only cells located within columnar boundaries were analyzed (i.e., septal cells were excluded). **B.** >1 prior Hit to CW increases CW-evoked responses. Conventions as in **Fig. 3D**. **C.** Quantification of trial history effects on CW responses (prior same 1 Hit vs prior NoGo: p = 0.55, prior same >1 Hit vs prior NoGo: p = 1e-4, prior different >1 Hit vs prior NoGo: p = 0.76, prior same >1 Hit vs prior different >1 Hit: p = 1e-4, permutation test). Conventions as in **Fig. 3E**. Related to **Figure 4** and **Figure S3**.

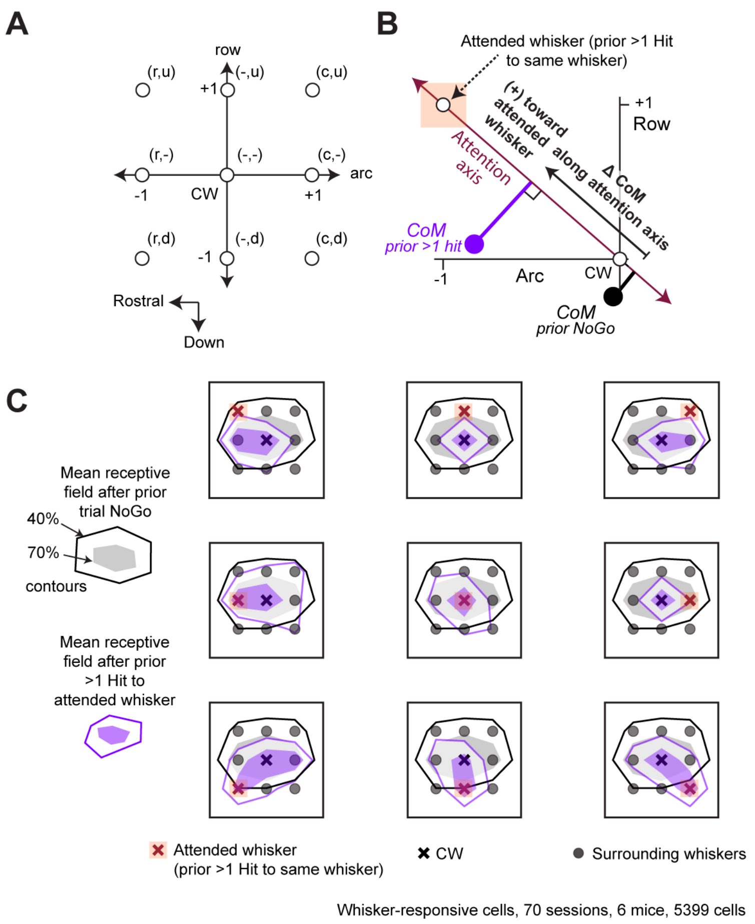

Figure S5. Additional analysis of receptive field shifts. A. Coordinate system for calculating tuning center-of-mass (CoM). The CW is at (0,0), and whiskers are separated by 1 unit in row or arc dimensions. **B.** Calculation of CoM tuning shift along the attention axis. The attention axis was defined as the line connecting the CW coordinate (0,0) with the coordinate of the attended whisker. The CoM measured after prior NoGo trials and after prior >1 hit to the attended whisker were projected onto this axis, and the CoM shift was calculated as distance along this axis, with positive values indicating shift toward the attended whisker. **C.** Mean receptive field for all cells imaged in the column corresponding to the center whisker in the piezo array (CW), either after prior trial NoGo (gray) or after >1 prior hits to an attended whisker (blue). The attended whisker is shown as the red x. The blue tuning functions are generally shifted toward the attended whisker. The blue tuning function in the center panel shows when the CW is attended. In this case, tuning sharpens around the CW. Receptive fields are shown as contour plots relative to maximum response. Related to **Figure 5**.

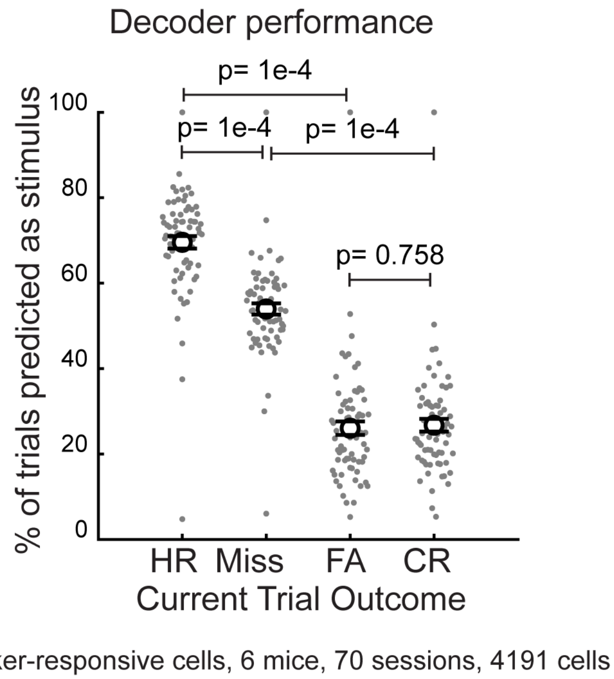

Figure S6. **Decoder performance analyzed separately for behavioral Hit, Miss, False Alarm, and Correct Reject trials.** Each point is the decoder for one imaging session, plotting decoder performance for detecting any whisker. Each decoder was trained on all trial types, but tested separately on held-out trials from the 4 trial types. Conventions as in **Fig. 6B**. P-values are for difference of means between indicated pairs of conditions (permutation test, alpha = 0.05). Related to **Figure 6**.

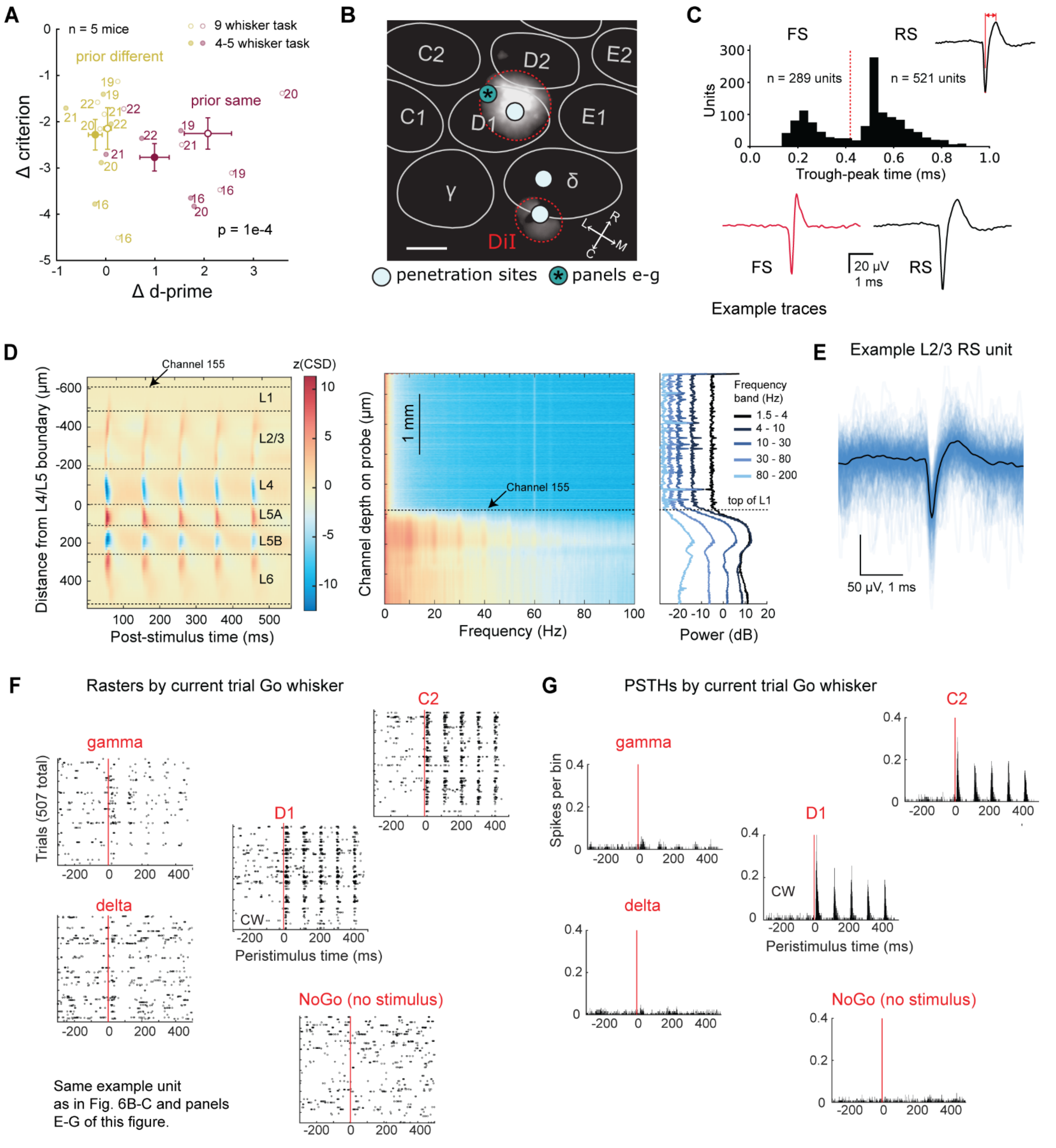

Figure S7. **Neuropixels recording methods. A.** Behavioral shifts in *d'* and *c* for mice that were initially trained on the 9-whisker task version, and then were tested either using 9 whiskers (open) or 4-5 whiskers only (filled). When performing the task with 4-5 whiskers, reward history still drives a whisker-specific Δ *d'* effect, but this is smaller than when performing with 9 whiskers (p = 1e-4, comparing same vs. different shift in Δ *d'* for 9-whisker task and 4-5 whisker task. Paired differences in same vs. different Δ *d'* shifts across session types were tested against zero). Numbered points are individual mice. Larger symbols are mean ± SEM across mice. **B.** Example histological reconstruction of a Neuropixels recording site to the D1 whisker column. Dashed circles are probe

tracks marked with 1,1′-Dioctadecyl-3,3,3′,3′-tetramethylindocarbocyanine perchlorate (DiI), outlines are from L4 barrels, white circles are reconstructed penetration sites. Asterisk marks the recording site for the example cell in **Fig. 6B** and in **S7** panel **E-G**. Scale bar = 100 μm. **C.** Classification of fast spiking (FS) and regular spiking (RS) units by trough-to-peak time in extracellular spike waveform. Bottom, example RS and FS spike waveform. **D.** Left, example current source density (CSD) analysis to identify laminar boundaries. See Methods for details. Right, power spectrum analysis to identify the top of L1, defined as the transition from high to low local field potential (LFP) power across a wide range of frequency bands. **E.** Mean spike waveform for the example L2/3 RS unit from **Fig. 6B**. Blue traces, individual spikes. **F.** Whisker-evoked spike rasters separated by current trial Go whisker (gamma, delta, D1, C2) or no whisker stimulus (NoGo trials), for the example L2/3 RS unit from **Fig. 6B**. All trial histories are combined in this plot. **G.** Whisker-evoked peristimulus time histogram (PSTH) for the same example unit as in panel **E**. Plotting conventions as in **Fig. 6C**. Related to **Figure 7**.

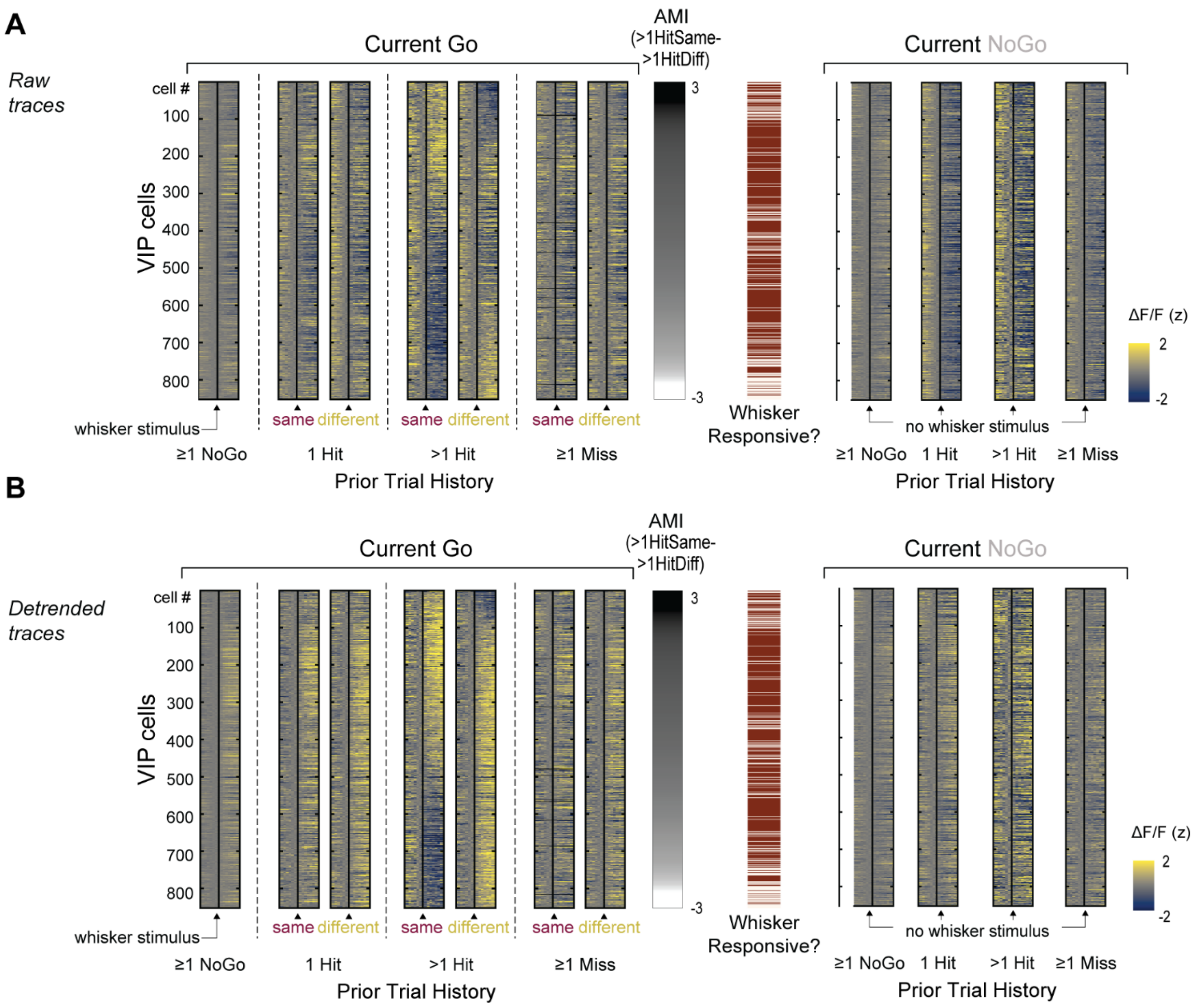

Figure S8. Additional analysis of attentional signals in VIP cells. A. Mean raw ΔF/F trace for each L2/3 VIP cell, sorted by $AMI_{>1HitSame->1HitDiff}$ for each cell. Cell order is the same across all columns in the panel. **B.** Mean detrended ΔF/F raw trace for each L2/3 VIP cell, sorted by $AMI_{>1HitSame->1HitDiff}$ for each cell. Related to **Figure 8**.
