# Supplementary material for: Reward history guides focal attention in whisker somatosensory cortex
Source: Nat Commun. 2025 Jul 1;16:5580. doi: 10.1038/s41467-025-60592-w (PMC12219055; doi:10.1038/s41467-025-60592-w)
Supplement: Supplementary file 2 — Reporting Summary [file 41467_2025_60592_MOESM2_ESM.pdf]

nature portfolio

Corresponding author(s): Daniel E. Feldman

Last updated by author(s): 05/18/2025

Reporting Summary

Nature Portfolio wishes to improve the reproducibility of the work that we publish. This form provides structure for consistency and transparency in reporting. For further information on Nature Portfolio policies, see our Editorial Policies and the Editorial Policy Checklist.

Please do not complete any field with "not applicable" or n/a. Refer to the help text for what text to use if an item is not relevant to your study.
For final submission: please carefully check your responses for accuracy; you will not be able to make changes later.

Statistics

For all statistical analyses, confirm that the following items are present in the figure legend, table legend, main text, or Methods section.

n/a	Confirmed	
☐	☒	The exact sample size (*n*) for each experimental group/condition, given as a discrete number and unit of measurement
☐	☒	A statement on whether measurements were taken from distinct samples or whether the same sample was measured repeatedly
☐	☒	The statistical test(s) used AND whether they are one- or two-sided<br>*Only common tests should be described solely by name; describe more complex techniques in the Methods section.*
☐	☒	A description of all covariates tested
☐	☒	A description of any assumptions or corrections, such as tests of normality and adjustment for multiple comparisons
☐	☒	A full description of the statistical parameters including central tendency (e.g. means) or other basic estimates (e.g. regression coefficient) AND variation (e.g. standard deviation) or associated estimates of uncertainty (e.g. confidence intervals)
☐	☒	For null hypothesis testing, the test statistic (e.g. *F*, *t*, *r*) with confidence intervals, effect sizes, degrees of freedom and *P* value noted<br>*Give P values as exact values whenever suitable.*
☒	☐	For Bayesian analysis, information on the choice of priors and Markov chain Monte Carlo settings
☒	☐	For hierarchical and complex designs, identification of the appropriate level for tests and full reporting of outcomes
☒	☐	Estimates of effect sizes (e.g. Cohen's *d*, Pearson's *r*), indicating how they were calculated

Our web collection on statistics for biologists contains articles on many of the points above.

Software and code

Policy information about availability of computer code

Data collection	Behavioral task control, user input and task monitoring were performed using custom Igor Pro 6.0 (WaveMetrics) routines and Arduino IDE 1.6.6. Imaging data collection was performed using ScanImage 5.6 (Vidrio Technologies) under the Matlab R2019b environment (MathWorks). Data collection for spike recordings was performed using SpikeGLX software release v.20201024 (http://billkarsh.github.io/SpikeGLX/), Imec phase30 v3.31. Behavioral movies used for DeepLabCut analyses were acquired using SpinView (Spinnaker SDK 3.0.0.118, Teledyne FLIR).
Data analysis	DeepLabCut (Mathis et al., 2018, https://github.com/DeepLabCut/DeepLabCut) was used for analysis of whisker/body motion and pupil size. Histological sections were manually aligned using Fiji (https://imagej.net/software/fiji/). Custom MATLAB pipeline code (Ramamurthy et al, 2023, https://github.com/dfeldman189/Ramamurthy2023Data; adapted from LeMessurier, 2019, https://github.com/alemessurier/imaging_analysis_pipeline) was used for processing of imaging data. Correction for slow XY drift was performed using dftregistration (https://www.mathworks.com/matlabcentral/fileexchange/18401-efficient-subpixel-image-registration-by-cross-correlation). Generalized linear models for neural decoding analyses were built using 'cvglmnet' in MATLAB. Spike sorting was performed using Kilosort3 (https://doi.org/10.5281/zenodo.10713583) followed by manual curation using the 'phy' GUI (https://github.com/kwikteam/phy). The LFP power spectrum was computed using 'lfpBandPower' (https://github.com/cortex-lab/neuropixels/). Original code for data analysis is available in the Zenodo repository, https://doi.org/10.5281/zenodo.14888799.

For manuscripts utilizing custom algorithms or software that are central to the research but not yet described in published literature, software must be made available to editors and reviewers. We strongly encourage code deposition in a community repository (e.g. GitHub). See the Nature Portfolio guidelines for submitting code & software for further information.

Data

Policy information about availability of data

All manuscripts must include a data availability statement. This statement should provide the following information, where applicable:

- Accession codes, unique identifiers, or web links for publicly available datasets
- A description of any restrictions on data availability
- For clinical datasets or third party data, please ensure that the statement adheres to our policy

The behavior data, 2p imaging data and extracellular recording data generated in this study have been deposited in the Zenodo repository, under accession code https://doi.org/10.5281/zenodo.14888799.

Research involving human participants, their data, or biological material

Policy information about studies with human participants or human data. See also policy information about sex, gender (identity/presentation), and sexual orientation and race, ethnicity and racism.

Reporting on sex and gender	The study did not involve human participants or human data.
Reporting on race, ethnicity, or other socially relevant groupings	The study did not involve human participants or human data.
Population characteristics	The study did not involve human participants or human data.
Recruitment	The study did not involve human participants or human data.
Ethics oversight	The study did not involve human participants or human data.

Note that full information on the approval of the study protocol must also be provided in the manuscript.

Field-specific reporting

Please select the one below that is the best fit for your research. If you are not sure, read the appropriate sections before making your selection.

☒ Life sciences ☐ Behavioural & social sciences ☐ Ecological, evolutionary & environmental sciences

For a reference copy of the document with all sections, see nature.com/documents/nr-reporting-summary-flat.pdf

Life sciences study design

All studies must disclose on these points even when the disclosure is negative.

Sample size	At least 6 mice were used for each type of imaging experiment, and at least 3 mice for Neuropixels recordings, following standard mouse n's for this type of study in the literature. Because the magnitude and variance of these effects were not known before this study, we could not use power analysis to precalculate sample sizes.
Data exclusions	For each behavioral session, we excluded early and late low-performance epochs (d'<0.5) that reflect motivational effects were excluded. Trials in which any licks occurred during the post-stimulus window (0 – 0.799 s) were excluded from analysis. Cells were excluded from analyses if the columnar whisker (CW) of the cell was not included among the 9 whisker stimuli tested during a given session, which indicates that the imaging field was mistargeted. These exclusions are described in the manuscript. Two mice used for extracellular recordings were not included in analyses of spike data due to mistargeting of the Neuropixels probe.
Replication	Behavioral effects of attention guided by reward history were replicated across individual mice in standard 9 whisker task and 4-5 whisker task sessions (Fig.1, Fig. S1) and Botox sessions (Fig. 2). Attentional boosting of PYR cell activity is replicated across individual mice (Fig. 3).
Randomization	Mice were randomly allocated for data collection in imaging experiments and spike recordings, other than approximately matching the total numbers of male and female animals used in the study. The whisker identity on each Go trial was randomly selected from nine possible whiskers, therefore allocation of trials to "prior same whisker" and "prior different whisker" conditions was also randomized in all analyses.
Blinding	All analyses were automated and performed using the identical procedures across all conditions. The investigator could not be blinded to the status of Botox injection in behavioral sessions because absence of whisker movements following Botox injection was clearly visible, however analyses were automated and performed using procedures identical to standard sessions.

Reporting for specific materials, systems and methods

We require information from authors about some types of materials, experimental systems and methods used in many studies. Here, indicate whether each material, system or method listed is relevant to your study. If you are not sure if a list item applies to your research, read the appropriate section before selecting a response.

Materials & experimental systems

n/a	Involved in the study	
☒	☐	Antibodies
☒	☐	Eukaryotic cell lines
☒	☐	Palaeontology and archaeology
☐	☒	Animals and other organisms
☒	☐	Clinical data
☒	☐	Dual use research of concern
☒	☐	Plants

Methods

n/a	Involved in the study	
☒	☐	ChIP-seq
☒	☐	Flow cytometry
☒	☐	MRI-based neuroimaging

Animals and other research organisms

Policy information about studies involving animals; ARRIVE guidelines recommended for reporting animal research, and Sex and Gender in Research

Laboratory animals	The study used 22 adult mice (transgenic strains of Mus musculus). These included 7 Drd3-Cre;Ai162D mice and 10 VIP-Cre;Ai162D mice (used for behavior and 2-photon imaging), and 5 offspring from Drd3-Cre x Ai162D crosses (genotype not determined) used in extracellular recording experiments. VIP-Cre (JAX # 10908) and Ai162D mice (JAX # 031562) were from The Jackson Laboratory. Drd3-Cre mice were from Gensat MMRRC (strain number 034610). Mice of all strains were 2-3 months old at surgery. Mice were kept in a reverse 12:12 light cycle, and were housed with littermates before surgery and individually after cranial window surgery (ambient temperature: 20-26°, humidity: 30-70%). Mice were roughly evenly divided between male and female, and no sex differences were found for the results reported here.
Wild animals	The study did not involve wild animals.
Reporting on sex	All key results were verified using a linear mixed-effects model, in which mouse sex was modeled as a fixed effect and found to be not significant.
Field-collected samples	The study did not involve samples collected from the field.
Ethics oversight	All methods followed NIH guidelines and were approved by the UC Berkeley Animal Care and Use Committee.

Note that full information on the approval of the study protocol must also be provided in the manuscript.

Plants

Seed stocks	The study did not involve plants.
Novel plant genotypes	The study did not involve plants.
Authentication	The study did not involve plants.

This checklist template is licensed under a Creative Commons Attribution 4.0 International License, which permits use, sharing, adaptation, distribution and reproduction in any medium or format, as long as you give appropriate credit to the original author(s) and the source, provide a link to the Creative Commons license, and indicate if changes were made. The images or other third party material in this article are included in the article's Creative Commons license, unless indicated otherwise in a credit line to the material. If material is not included in the article's Creative Commons license and your intended use is not permitted by statutory regulation or exceeds the permitted use, you will need to obtain permission directly from the copyright holder. To view a copy of this license, visit http://creativecommons.org/licenses/by/4.0/
